# 

**Published:** 1800-09

**Authors:** 


NEW MEDICAL PUBLICATIONS IN AUGUST.

New Inventions and new Directions to the Ruptured, by a private Gentleman. Low.

A Treatiſe on the Chemical Hiſtory and Medical Powers of ſome of the moſt celebrated Mineral Waters, with Practical Remarks on the Aqueous Regimen. To which are added, Obſervations on the Uſe of Cold and Warm Bathing, by William Saunders, M. D. 8s. boards. Phillips, George Yard.

A Practical Treatiſe on the different Fevers of the Weſt Indies, and their diagnoſtic Symptoms, by William Fowle, M. D. 2s. 6d. H. D. Symonds.

NEW MEDICAL PUBLICATIONS IN GERMANY.

*Ueber die Pulſader Jeſhwulſte*, i. e. On Aneuriſms and their Surgical Treatment; by Dr. AYRER, with a plate, pp. 387, 8vo. Gottingen, 1800, Dieterich.

*Beitrag zur Beurtheilung*, i. e. Contribution to the Criticiſm of the Brunonian Syſtem of Medicine; by LEWIS CHRIST. WILLIAM CAPPEL, Doctor of Medicine at Gottingen, ſecond edition, much altered, pp. 448, 8vo. 1800. Gottingen, Dieterich.

*Ritter's Darſtellung*, i. e. RITTER's Hiſtory of the lateſt Inquiries relating to the Illumination of Phoſphorus in Azote, and of the Reſults of it for Chemical Theory, 8vo. 1800. Jena, Fromman.

*G. J. Beer's Methode*, i. e. Method of extracting the Cataract along with the Capſula; with ſome other improvement of the operations of the Cataract in general. 1799, pp. 60, 8vo. with a plate. Vienna, Schaumburg.

---

### To CORRESPONDENTS.

*Our Correſpondents are particularly requeſted to give the* TITLES *of their papers as well as the* Title of the general Subject.

*We have received friendly and wholeſome advice, which we hope to profit by, from an anonymous Correſpondent reſpecting controverſial papers, and thoſe of young authors; but we ſhould have been more obliged to him if he had given us his addreſs, as we might then have availed ourſelves of the advantage of his opinion on other occaſions.*

*Communications have been received from Meſſrs. Malin, Favell, Clutterbuck, Ballard, Thackeray, Groſe, Bellamy, and S. S. Doctors Curſon, Gilleſpe, and Simons.*

